# Esophageal Cancer-Derived Extracellular Vesicle miR-21-5p Contributes to EMT of ESCC Cells by Disorganizing Macrophage Polarization

**DOI:** 10.3390/cancers13164122

**Published:** 2021-08-16

**Authors:** Jing Song, Peiyan Yang, Xiuwen Li, Xinyi Zhu, Mengxin Liu, Xuexin Duan, Ran Liu

**Affiliations:** 1 Key Laboratory of Environmental Medicine Engineering, Ministry of Education, School of Public Health, Southeast University, Nanjing 210009, China; songjingseu@163.com (J.S.); fydyangpy@126.com (P.Y.); flwzypyy@yeah.net (M.L.); 2 The Affiliated Zhongda Hospital, Medical School of Southeast University, Nanjing 210009, China; lixiuwen2006@126.com (X.L.); china_nj_zxy@sina.com (X.Z.); 3 State Key Laboratory of Precision Measuring Technology & Instruments, College of Precision Instrument and Opto-electronics Engineering, Tianjin University, Tianjin 300072, China; xduan@tju.edu.cn

**Keywords:** esophageal squamous cell carcinoma (ESCC), EVs-miR-21-5p, macrophage polarization, epithelial-mesenchymal transition

## Abstract

**Simple Summary:**

Macrophage polarization-associated extracellular vesicles (EVs) play crucial roles in tumor progression. The role of miR-21-5p in EVs during esophageal squamous cell carcinoma (ESCC) development must be clarified. This study aimed to identify the relationship between ESCC cells and macrophages in different polarization states during the delivery of EVs-miR-21-5p. We found that M0 macrophages took up overexpressed EVs-miR-21-5p secreted by EC109 or EC9706 cells, which transformed them into M2 macrophages through the PTEN/AKT/STAT6 pathway. This, in turn, contributed to secretion of high levels of TGF-β1 by M2 macrophages and promoted esophageal cancer cell epithelial-mesenchymal transition via the TGF-β/Smad2 axis. These findings indicate that EVs-miR-21-5p may be a critical molecule for ESCC.

**Abstract:**

The disorganized polarization of tumor-associated macrophages (TAMs) exerts a critical effect on tumor progression. MicroRNAs (miRNAs) in extracellular vesicles (EVs) secreted from cancer cells may contribute to this process. However, the relationship between TAMs and EVs-miRNAs-mediated regulation in esophageal squamous cell carcinoma (ESCC) remains unclear. In the present study, immunoaffinity magnetic beads combined with antiepithelial cell adhesion molecules (EpCAM) were used to isolate and identify EVs-miR-21-5p from the plasma of ESCC patients. An in vitro coculture system was designed to evaluate the effect of esophageal cancer cells with miR-21-5p overexpression on macrophage polarization. We found that phorbol myristate acetate-induced THP-1 macrophages took up EVs-miR-21-5p from EC109 or EC9706 cells and were transformed into M2 macrophages. This, in turn, contributed to the excessive migration and invasion of esophageal cancer cells. The mechanism underlying these changes may involve activation of M2 macrophages by upregulated ESCC-derived EVs-miR-21-5p through the PTEN/AKT/STAT6 pathway. This may result in esophageal cancer cell epithelial-mesenchymal transition (EMT) via TGF-β/Smad2 signaling. Our results indicate positive feedback between M2 macrophage polarization and EMT of esophageal cancer cells in the tumor microenvironment via shuttling of miR-21-5p in tumor-derived EVs.

## 1. Introduction

Esophageal cancer, a malignancy with high incidence and mortality, is a serious threat to human life and health. According to 2020 global cancer statistics, esophageal cancer ranks seventh in global cancer incidence (604,000 new cases) and sixth in total mortality (544,000 deaths). It is estimated that one in every 18 deaths from cancer is from esophageal cancer [1]. In China, esophageal squamous cell carcinoma (ESCC) accounts for approximately 90% of the overall incidence of esophageal cancer and nearly half of all esophageal cases worldwide [2]. Notably, the early symptoms of esophageal cancer are atypical and difficult to identify, which leads to late diagnoses and partly explains the high mortality and poor prognosis [3]. Therefore, identifying targets that would permit early diagnosis of esophageal squamous cell carcinoma (ESCC) is crucial [4]. 

One potential source of such biomarkers is the tumor microenvironment (TME), which contains tumor cells and several stromal cell types, including tumor-associated macrophages (TAMs), that orchestrate various factors to facilitate tumor progression [5]. According to their functions and the presence of surface markers, macrophages can be classified into classically activated M1 macrophages and alternatively activated M2 macrophages. M1 macrophages are activated by cytokines, such as interferon-γ, and predominantly secrete proinflammatory and immunostimulatory cytokines, such as IL-6 and TNF-α. Most TAMs more closely resemble M2 macrophages activated by Th2 cytokines, such as IL-4, IL-10, and IL-13 [6,7], but evidence indicates that there are two populations of TAMs: M1-TAMs and M2-TAMs [8]. Studies indicate that M2-TAMs promote tumor proliferation, invasion, metastasis, and angiogenesis, resulting in the angiogenesis of tumor cells [9,10,11]. While M2-TAMs have been well studied, less is known about the significance of M1-TAMs, and the relationship between M1 and M2 macrophages in the TME may mediate tumor occurrence and development.

A key component of cancer-related cells, including macrophages, is the extracellular vesicle (EV). Increasing numbers of studies have shown that tumor-derived EVs widely participate in the process of material and message exchange between tumor cells and their surrounding cells, and that these exchanges play key roles in the development of various tumors [12,13,14]. In addition, abnormal levels of miRNA molecules within EVs (EVs-miRNAs) in TME cells may result in dysfunction of the epithelial-mesenchymal transition (EMT) [15]. For example, Madhavan, et al. [16] found that miR-1246, miR-4644, miR-3976, and miR-4306 levels in plasma EVs were significantly increased in patients with pancreatic cancer, contributing to the poor diagnosis of this disease. Similarly, Sugimachi, et al. [17] compared EVs-miRNA levels in liver cancer patients and healthy controls and found that miR-718 was significantly downregulated in the plasma EVs of liver cancer patients. Therefore, it is likely that EVs, as key effectors of cellular communication in the TME, play critical roles in tumorigenesis, angiogenesis, immune escape, drug resistance, and metastasis [18,19].

Our group previously demonstrated that miR-21-5p is upregulated in EVs of human esophageal cancer cells, and that this upregulation is related to the poor prognosis of esophageal cancer [20]. However, the underlying mechanisms by which miR-21-5p in tumor-derived EVs impacts the progression of esophageal cancer are unknown. In the present study, immunoaffinity magnetic beads combined with an antiepithelial cell adhesion molecule (EpCAM) antibody were used to isolate tumor-derived EVs from plasma samples. We found that the level of miR-21-5p in tumor-derived EVs from the plasma of esophageal cancer patients was higher than that of healthy control patients. The role of EVs-miR-21-5p on the polarization of macrophages was identified. We found that EVs-miR-21-5p secreted by EC109 was ingested by M0 macrophages, which contributed to the polarization of M2 macrophages. Interestingly, this dysregulated balance of macrophage polarization promoted the excessive migration and invasion of esophageal cancer cells. This finding further elucidates the pathogenesis of ESCC.

## 2. Results

### 2.1. Upregulation of Tumor-Derived EVs-miR-21-5p Correlates with ESCC Progression

**Figure 1:**
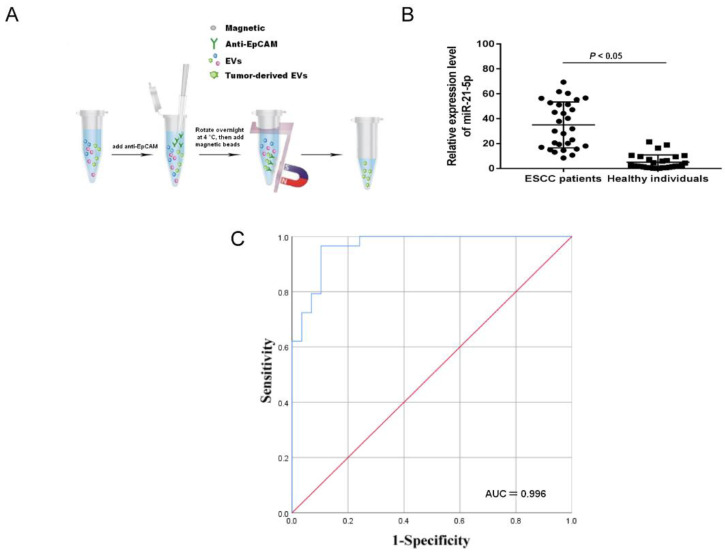
The expression of EVs-miR-21-5p in ESCC plasma. (**A**) Scheme of isolation of tumor-derived EVs from the plasma with anti-EpCAM. (**B**) The expression of tumor-derived EVs-miR-21-5p. (**C**) ROC curve analysis of miR-21-5p. EpCAM, epithelial cell adhesion molecule.

To evaluate the level of miR-21-5p in plasma EVs, EpCAM, a multifunctional transmembrane protein involved in the regulation of tumor cell adhesion, proliferation, migration, stemness and EMT, was used as a marker to identify and isolate tumor-derived EVs [21]. The use of this marker partially avoided the collection of EVs secreted by nontumor cells present in plasma (Figure 1A). Compared to the healthy group, miR-21-5p was significantly overexpressed in the plasma EVs of ESCC patients (Figure 1B, *p* < 0.05). Conditional logistic regression demonstrated that elevated EVs-miR-21-5p in ESCC patients was positively correlated with esophageal cancer risk (Table 1, odds ratio [OR] = 1.34, *p* < 0.001).

To explore the diagnostic power of tumor-derived EVs-miR-21-5p for esophageal cancer, an ROC analysis was used to analyze the sensitivity and specificity of the levels of EVs-miR-21-5p in this application (Figure 1C).

The area under the curve of tumor-derived EVs-miR-21-5p was 0.966, and the sensitivity and specificity were 96.6% and 89.7%, respectively (Table 2). These results indicate that tumor-derived EVs-miR-21-5p may play a critical role in ESCC progression.

### 2.2. EVs-miR-21-5p Secreted by ESCC Cells Are Taken Up by PMA-Treated Macrophages

**Figure 2:**
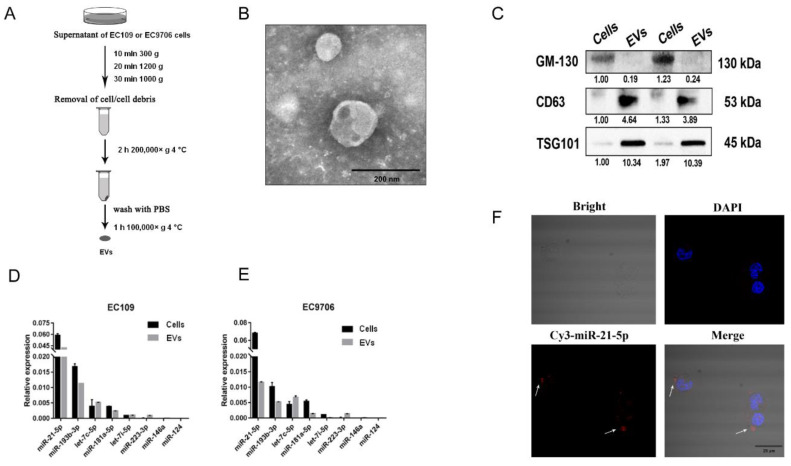
EVs-miR-21-5p secreted by ESCC cells were taken up by PMA-treated macrophages. (**A**) Workflow describing isolation of EVs from EC109 supernatants. (**B**) Morphology of EVs under a transmission electron microscope (scale bar, 200 nm). (**C**) Western blot analysis of EVs markers (CD63 and TSG-101) and a protein, GM-130, that is not found in EVs. Macrophage polarization-related miRNAs were detected by q-RT-PCR in EC109 (**D**) and EC9706 (**E**) cells. (**F**) Confocal microscopy was used to observe the uptake of Cy-3 labeled EVs-miR-21-5p by PMA-treated macrophages. Blue: DAPI staining; red: Cy3-miR-21-5p; scale bar: 25 μm. The uncropped Western Blot images can be found in Supplementary Materials.

Multiple studies indicate that the contents of secretion from EVs differ among cancer cells [22,23]. To obtain EVs from ESCC cells, a differential centrifugation method was used to isolate the EVs of EC109 and EC9706 cells (Figure 2A). Then, the EVs were observed by transmission electron microscopy. We found that most EVs were round or elliptical with a diameter ranging between 30 and 100 nm, and they displayed a complete membrane structure with low-density content (Figure 2B). 

In Western blot analyses, EVs-specific proteins CD63 and TSG101 were detected in the isolated EVs, whereas GM-130, which is expressed in ESCC but is not present in EVs, was not detected (Figure 2C). Several macrophage polarization-related miRNAs identified in our previous study were detected in EC109 and EC9706 cells [20]. These results confirmed the purity of the isolated EVs. Subsequent q-RT-PCR analyses suggested that miR-21-5p is the most highly expressed circulating miRNA in EC109 and EC9706 cells (Figure 2D,E).

To elucidate whether EVs-miR-21-5p secreted by ESCC cells can be transferred to macrophages, Cy3-labeled miR-21-5p mimics were transfected into EC109. After 48 h, the EVs isolated from the supernatants of these cells were incubated with phorbol myristate acetate (PMA)-treated THP-1 cells for 24 h. We found that Cy3-labeled miR-21-5p mimics were taken up by PMA-treated macrophages, and were mainly distributed in the cytoplasm of the recipient cells (Figure 2F). These observations indicated that EVs-miR-21-5p secreted by ESCC can be ingested by PMA-treated macrophages.

### 2.3. EVs-miR-21-5p Secreted by EC109 or EC9706 Cells Promote Polarization of M2 Macrophages

**Figure 3:**
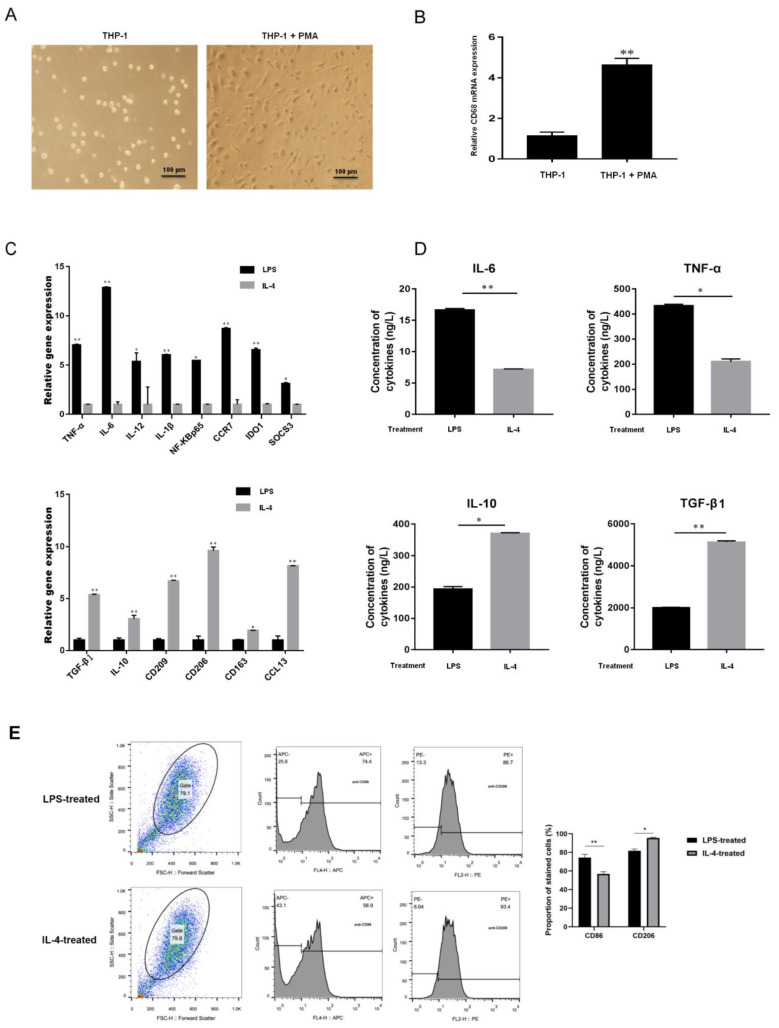
Identification of macrophages after M1- or M2-type polarization. (**A**) The morphology of THP-1 cells after PMA treatment (scale bar, 100 μm). (**B**) The mRNA expression of CD86 in PMA-treated THP-1 cells as determined by q-RT-PCR. (**C**) The mRNA expression of macrophage markers of M1 (IL-6, IL-12, TNF-α, IL-1β, NF-κB P65, CCR7, IDO1, and SOCS3) and M2 (CD206, CD209, CD163, CCL13, IL-10, and TGF-β1) as determined by q-RT-PCR. (**D**) The protein expression of the M1 and M2 macrophage markers IL-4, TNF-α, IL-10, and TGF-β1 were measured by ELISA. (**E**) Flow cytometric identification of LPS- and IL-4-treated macrophages. * *p* < 0.05, ** *p* < 0.01.

After treatment with 100 ng/mL PMA, THP-1 cells changed from a suspended morphology to an adherent morphology (Figure 3A), and the M0 macrophage marker CD68 was upregulated (Figure 3B). The adherent macrophages were incubated with LPS or IL-4 to promote differentiation into M1 or M2, respectively. Compared to IL-4-treated macrophages, the transcription levels of the M1 markers IL-6, IL-12, TNF-α, IL-1β, NF-κB P65, CCR7, IDO1, and SOCS3 were increased in the M1 macrophages induced by LPS, but the M2 macrophages markers CD206, CD209, CD163, CCL13, IL-10, and TGF-β1 were downregulated (Figure 3C). The levels of IL-4, IL-10, TNF-α, and TGF-β1 in the supernatants of LPS- or IL-4-treated adherent macrophages were measured by ELISA. The results showed that IL-6 and TNF-α were elevated in LPS-induced M1 macrophages, but that IL-10 and TGF-β1 were upregulated in IL-4-treated M2 macrophages (Figure 3D). In flow cytometric analyses, 74.4% of LPS-stimulated macrophages were positive for expression of CD86 (CD86+) and 86.7% were CD206+, whereas more than 56.9% of IL-4–stimulated macrophages were CD86+, and 93.4% were CD206+ (Figure 3E).

**Figure 4:**
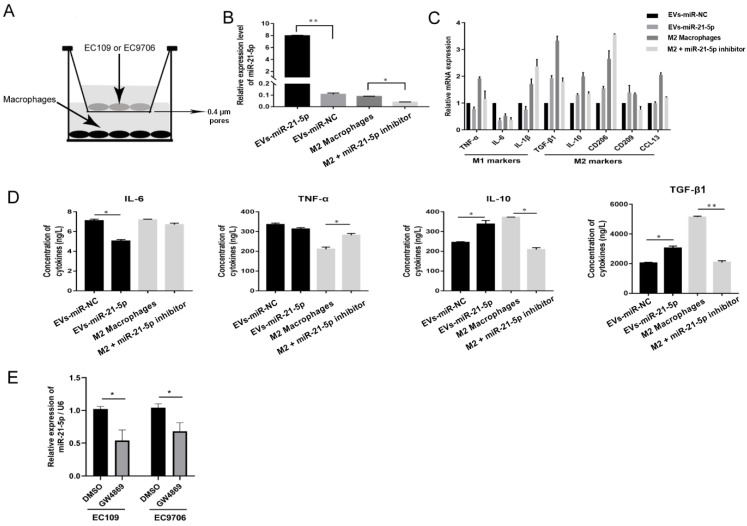
EVs-miR-21-5p promote M2 macrophage polarization. (**A**) Schematic design of the Transwell experiments. (**B**) Expression of miR-21-5p in macrophages in the coculture system as detected by q-RT-PCR. (**C**) The mRNA expression of M1 and M2 macrophage markers was detected by q-RT-PCR. (**D**) The protein expression of M1 and M2 macrophage markers IL-4, TNF-α, IL-10, and TGF-β were measured by ELISA. (**E**) Relative expression of miR-21-5p in the culture supernatant of EC109 and EC9706 treated with GW4869. * *p* < 0.05; ** *p* < 0.01. The *F*- and *t*-tests were applied to assess statistical significance in group comparisons. Error bars indicate mean ± SD. EVs-miR-NC, EC109 cells were transfected with miRNAs mimic-NC for 48 h and cocultured with PMA-treated macrophages for another 24 h. EVs-miR-21-5p, EC109 cells were transfected with miR-21-5p mimic for 48 h and cocultured with PMA-treated macrophages for another 24 h. M2 macrophage, 100 ng/mL PMA was added to THP-1 cells for 24 h, incubated with 20 ng/mL IL-4 for another 24 h, and transfected with miRNAs inhibitor-NC for 48 h. M2 macrophage + miR-21-5p inhibitor, M2 macrophage was transfected with miR-21-5p inhibitor for 48 h.

To further explore the role of EVs-miR-21-5p in macrophage polarization, an in vitro coculture system was designed to permit exploration of the molecular events that characterize the communication between EC109 and TAMs (Figure 4A). EC109 cells were transfected with a miR-21-5p mimic for 48 h and cocultured with PMA-treated macrophages for another 24 h. This process resulted in increased miR-21-5p, CD209, CD206, IL-10, CCL13, and TGF-β1 transcription levels and upregulated IL-10 and TGF-β1 protein expression in the PMA-treated macrophages (Figure 4B,C, *p* < 0.05). However, when IL-4-induced M2 macrophages were transfected with a miR-21-5p inhibitor for 48 h, we observed elevated transcription levels of the M1 markers TNF-α, IL-6, and IL-1β and protein expression of IL-6 and TNF-α (Figure 4D, *p* < 0.05). 

To explore whether ESCC secreted extracellular miR-21-5p is blocked by vesicle inhibitors, 10 µM GW4869 was added to EC109 and EC9706 for 24 h. miR-21-5p expression in culture supernatants was downregulated with GW4869 treatment (Figure 4E). These results indicate that most miR-21-5p in supernatants was mainly derived from EVs.

### 2.4. EVs-miR-21-5p Activate Polarization of M2 Macrophages via the PTEN/PI3K/AKT/STAT6 Axis

To assess the potential mechanisms explaining the role of EVs-miR-21-5p in macrophage polarization, the TargetScan database was used to identify relevant targets of miR-21-5p. Among the potential targets of miR-21-5p identified in this way, PTEN stands out as an important contributor to the migration and invasion of cancer cells [24,25]; however, its roles in the TME and the polarization of TAMs remain unclear. 

**Figure 5:**
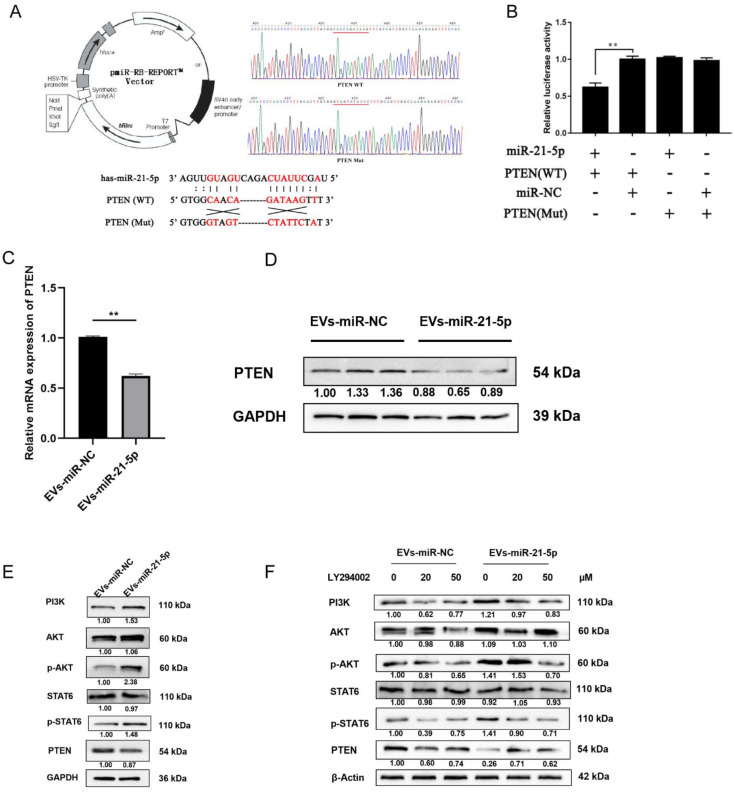
EVs-miR-21-5p activates M2 macrophage polarization via the PTEN/PI3K/AKT/STAT6 axis. (**A**) Plasmid vector pattern and region binding site of PTEN. The presence of the mutation in the 3′UTR was confirmed via DNA sequencing (**B**) THP-1 cells were respectively transfected for 48 h with pCDNA-PTEN-WT and pCDNA-PTEN-Mut or with miR-21-5p mimics and mimics-NC. The mRNA (**C**) and protein (**D**) levels of PTEN in EVs-miR-21-5p-treated macrophages. (**E**) Western blotting analysis of PI3K, STAT6, p-STAT6, Akt, p-Akt, and PTEN expression in PMA-treated THP-1 cells transfected with EVs-miR-21-5p. (**F**) Relative protein levels of the PTEN/PI3K/AKT/STAT6 axis after LY294002 treatment. The relative protein level was normalized to that of β-actin or GAPDH. The *F*- or *t*-test was applied to assess statistical significance in group comparisons; ** *p* < 0.01. Error bars indicate mean ± SD. EVs-miR-NC, EC109 cells were transfected with miRNAs mimic-NC for 48 h and cocultured with PMA-treated macrophages for another 24 h. EVs-miR-21-5p, EC109 cells were transfected with miR-21-5p mimic for 48 h and cocultured with PMA-treated macrophages for another 24 h. The uncropped Western Blot images can be found in Supplementary Materials.

To provide a biological test of the role of PTEN in this process, we transfected THP-1 cells with miR-21-5p mimics and vectors with wild-type or mutant versions of the 3′UTR of the *PTEN* gene to conduct a luciferase reporter assay (Figure 5A). Compared to control cells, the activity of the wild-type 3′UTR was suppressed by miR-21-5p overexpression. There was no significant change in this activity in cells expressing the mutant 3′UTR that were transfected with miR-21-5p mimics (Figure 5B, *p* < 0.05). These results indicated that miR-21-5p can bind to the 3′UTR of PTEN. In addition, PTEN expression was measured in cocultured macrophages. Here, miR-21-5p overexpression in EC109 cells suppressed PTEN expression at mRNA and protein levels (Figure 5C,D). 

Notably, a previous study indicated that inhibition of PTEN may regulate the polarization of M2 macrophages via activation of PI3K/Akt/STAT6 signaling in the progression of liver fibrosis [26]. Therefore, we speculated that the binding of miR-21-5p to *PTEN* mRNA and the resulting decreased expression of the PTEN enzyme may activate PI3K/Akt/STAT6 signaling in the progression of macrophage polarization. To identify whether PI3K/Akt/STAT6 signaling was mediated by miR-21-5p in TAMs, PI3K, Akt, p-Akt, STAT6, p-STAT6, and PTEN protein levels were detected by Western blot. Compared to the control group, PTEN expression was downregulated in M2 macrophages that had taken up EVs-miR-21-5p, and PI3K, p-Akt, and p-STAT6 levels were elevated (Figure 5E). When the PI3K inhibitor LY294002 (0, 20, and 50 µM) was added to the culture medium, the LY294002 resulting decreased expression of p-Akt and p-STAT6 in cocultured macrophages (Figure 5F). These results indicate that EVs-miR-21-5p may activate PI3K/Akt/STAT6 signaling by suppressing PTEN expression, and this mechanism may contribute to M2 macrophage polarization.

### 2.5. EVs-miR-21-5p Regulates Migration, Invasion, and Expression of EMT-Related Genes in EC109 and EC9706 Cells

**Figure 6:**
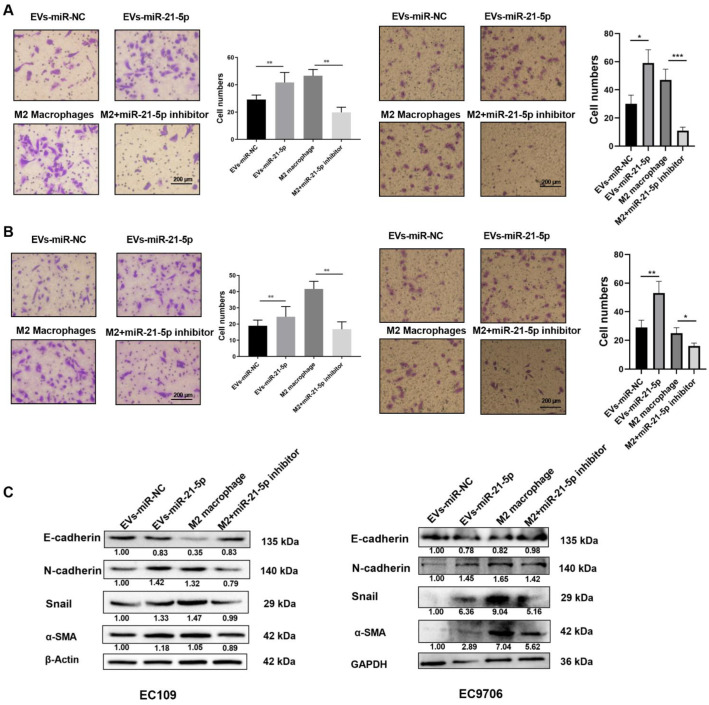
EVs-miR-21-5p regulates migration, invasion, and expression of EMT-related genes in EC109 and EC9706 cells. Representative images of a migration (**A**) and invasion (**B**) assay of EC109 and EC9706 cells that were cultured with the supernatants of miR-21-5p-overexpressing M0 macrophages or IL-4-induced macrophages transfected with a miRNA-21-5p inhibitor. (**C**) Western blotting analysis of N-cadherin, E-cadherin, Snail and α-SMA expression in EC109 and EC9706 cells. Values are expressed as means ± SD. The relative protein levels were normalized to β-actin or GAPDH. The *F* test was applied to assess statistical significance in group comparisons; * *p* < 0.05; ** *p* < 0.01, *** *p* < 0.001. Error bars indicate mean ± SD. The uncropped Western Blot images can be found in Supplementary Materials.

To identify the effect of M2 macrophages induced by EVs-miR-21-5p on the progression of ESCC cells, the supernatants of macrophages were added to ESCC cells for 24 h. The levels of migration, invasion, and EMT markers (N-cadherin, α-SMA, and Snail) were significantly elevated in EC109 or EC9706 cells that were cultured with the supernatant collected from miR-21-5p-overexpressing macrophages (Figure 6A–C). However, when we treated EC109 or EC9706 with the IL-4-induced M2 macrophage supernatant in the presence of a miR-21-5p inhibitor, EC109 and EC9706 migration, invasion, and EMT markers were downregulated. These observations indicate that EVs-miR-21-5p regulates the migration, invasion, and expression of EMT-associated genes in EC109 and EC9706 cells.

### 2.6. M2 Macrophage-Derived EVs-miR-21-5p Mediates the Expression of EMT-Associated Genes in Esophageal Cancer Cells through Modulation of TGF-β Signaling

**Figure 7:**
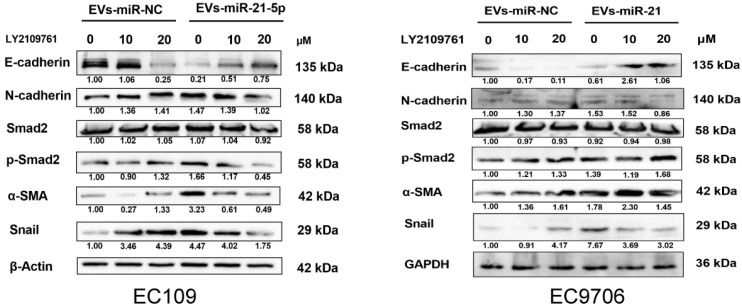
EVs-miR-21-5p derived from M2 macrophages promotes esophageal cancer cell EMT through TGF-β signaling. Western blotting analysis of E-cadherin, N-cadherin, α-SMA, Snail, Smad2, and p-Smad2 expression in EC109 or EC9706 at different dosages of LY210976 (0, 10, and 20 µM). The relative protein level was normalized to β-actin or GAPDH.

An increasing number of studies have shown that TGF-β signaling may play a key role in the EMT of various cancer cells [27,28]. To identify the potential mechanisms by which M2 macrophages enhance ESCC progression, EMT marker proteins were detected by Western blot. Compared to EC109 or EC9706 treated with the supernatant of PMA-induced macrophages, the level of N-cadherin, α-SMA, Snail, and p-Smad2 in EC109 or EC9706 treated with the supernatant of miR-21-5p overexpressing M0 macrophages, were significantly increased, while the expression of the epithelium marker E-cadherin was decreased. We used the TGF-β signaling inhibitor LY2109761 (10 and 20 µM) to identify whether blocking the TGF-β signaling pathway affected the EMT of EC109 and EC9706 cells. The results showed that N-cadherin, α-SMA, Snail, and p-SMAD2 decreased, while the E-cadherin protein level was dose-dependently upregulated in EC109 or EC9706 treated with LY2109761 (Figure 7). The above results indicated that M2 macrophage-derived EVs-miR-21-5p may regulate the expression of EMT-associated genes in esophageal cancer cells through the TGF-β signaling pathway. 

## 3. Discussion

**Figure 8:**
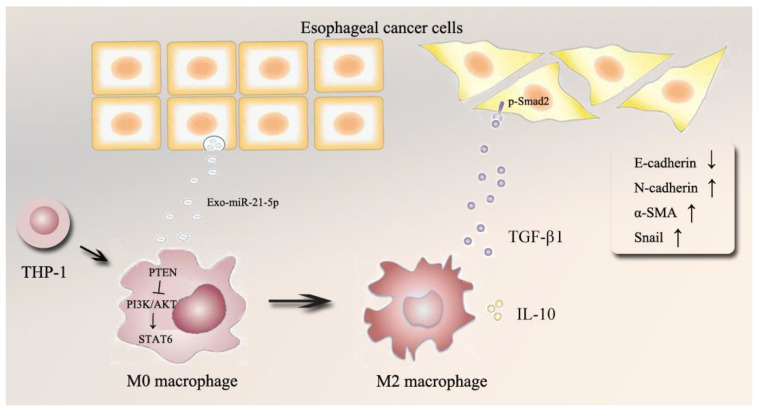
Schematic model of this study.

The TME, a dynamic system mediated by intercellular communication, plays a vital role in cancer progression. EVs participate in the interaction between tumors and macrophages [14,29]. Increasing evidence suggests that disorganized TAMs play a critical role in tumor progression, thus implicating mechanisms of communication in cancer development. EVs-miRNAs secreted from cancer cells may contribute to this process. However, the relationship between TAMs and regulation mediated by EVs-miRNAs in ESCC remains unclear [16,30]. Here, immunoaffinity magnetic beads combined with anti-EpCAM were used to isolate and identify the level of EVs-miR-21-5p from the plasma of ESCC patients. An in vitro coculture system was designed to evaluate the effects of esophageal cancer cells that overexpress miR-21-5p on macrophage polarization. We found that upregulated ESCC-derived EVs-miR-21-5p activated M0 macrophages to M2 macrophages through the PI3K/AKT/STAT6 pathway, which in turn resulted in high-level TGF-β1 secretion by M2 macrophages and changes of expression of EMT-associated genes in esophageal cancer cells via TGF-β/Smad2 signaling. We revealed a positive feedback mechanism between M2 macrophage polarization and EMT of esophageal cancer cells in the tumor microenvironment via the shuttling of tumor-derived EVs-miR-21-5p (Figure 8).

Our method for the isolation of the EVs secreted by tumor cells may contribute to the study of tumorigenesis and development. The Epithelial cell adhesion molecule (EpCAM) is expressed at basal levels in the normal epithelial cells but is upregulated in solid epithelial cancers [31]. Several studies have indicated that EpCAM plays critical roles in the migration, proliferation, and differentiation of tumor cells [32]. Moreover, EpCAM can be detected in the bodily fluid of cancer patients, suggesting that EpCAM is a biomarker for a variety of cancers [21,33]. Meanwhile, other studies have indicated that EpCAM protein exists in EVs, and EpCAM-positive EVs may be useful biomarkers in ovarian and pancreatic cancers [34,35]. Therefore, we used EpCAM expression as a marker for EVs secreted by tumor cells. The amount of precipitated EpCAM positive EVs in tumor patient plasma was more than that from healthy controls. Because we used U6 to quantitatively normalize miRNA levels, we were able to calculate the relative expression of miRNAs in EpCAM-positive EVs from the plasma of the tumor group and the healthy group. While EVs isolated from the body fluids of ESCC patients were useful for the study of ESCC, due to tumor heterogeneity, EVs from the culture medium of EC109 and EC9706 may lead to more reproducible studies of the shuttling of miR-21-5p between tumor cells and macrophages. 

A previous study indicated that isolation of the EVs from cells that were pretreated with fluorescently labeled miRNA mimics was an efficient method to demonstrate that miRNA from EVs can be phagocytized by other cells [36]. Therefore, to elucidate whether EVs-miR-21-5p secreted by ESCC cells can be transferred to macrophages, we used EC109 cells transfected with Cy3-labeled miR-21-5p mimics. The EVs isolated from the EC109 supernatant were incubated with PMA-treated THP-1 cells for 24 h. These EVs highly expressed total miR-21-5p, and part of this miR-21-5p was fluorescently labeled with Cy3. This labeling allowed us to observe the uptake of EVs by macrophages via laser confocal microscopy. In addition, packaging of miR-21-5p using EVs is also useful in the exploration of the effect of EVs-miR-21-5p on single cells. This method may not, however, allow the mimicking of the information transmission between esophageal cancer cells and macrophages by EVs.

In previous studies, the optimum concentration of LY294002 for the inhibition of PI3K in THP-1 cells was 10 µM [37], and in EC109 or EC9706 cells was 20 µM [38]. However, when we added 10 µM LY294002 to PMA-induced M0 macrophages that were cocultured with EC109 or EC9706 cells in a Transwell system, no significant changes to the phosphorylation of proteins associated with PI3K signaling were observed in M0 macrophages. When we used different concentrations of LY294002 (20, 30, 40, 50, and 60 µM) in the Transwell system, we found that the PI3K/Akt/STAT6 pathway was inhibited in the presence of LY294002 (20, 30, 40, and 50 µM). Therefore, we chose experiments using 20 and 50 µM LY294002 as the representative results in this study. 

Many signaling pathways related to macrophage polarization are reportedly crucial to the clarification of disease pathogenesis. The dysfunction of PTEN enhances the phosphorylation of Akt during M2 macrophage polarization, which contributes to lung cancer and hepatocellular carcinoma development [15,26]. The roles of the miR-21-5p, PTEN, and PI3K-AKT signaling pathways in the progression of ESCC remain unclear. We found that EVs could deliver miR-21-5p from esophageal cancer cells to macrophages, which activates M2 macrophage polarization by targeting PTEN. In addition, treatment with LY294002 significantly inhibited PI3K activity. Altered PTEN and miR-21-5p in macrophage polarization may thus influence the activity of PI3K/Akt pathway molecules via regulation of activities of PTEN, AKT and STAT6. 

Although the effects of M2 macrophages on immunosuppression and anti-inflammation have been demonstrated in previous studies [39,40], the mechanisms by which TGF-β1 secreted by M2 macrophages promote the progression of ESCC has remained unclear. Long-term stimulation by cancer cells may lead to disorganized macrophage polarization and excessive TGF-β1 levels. The process of tumor cell proliferation, differentiation, and apoptosis is regulated by TGF-β1, a key mediator in EMT during tumor formation. Many studies have demonstrated that endogenous TGF-β1 contributes to EMT through the TGFβ signaling pathway, which indicates that the inhibition of the TGF-β1 signaling pathway may be an effective cancer treatment. To investigate whether M2 TAM promoted the EMT process in esophageal cancer, the levels of E-cadherin, N-cadherin, Snail, and α-SMA were measured in EC109 or EC9706 treated with a coculture system. The results showed that EC109 or EC9706-treated M2 macrophages regulated ESCC cells EMT-related proteins via TGF-β signaling pathways.

## 4. Materials and Methods

### 4.1. Clinical Samples and Ethical Statement 

Blood samples were collected from 36 ESCC patients from Huaian Hospital, Huaian, China and from 36 healthy age and gender-matched individuals with no history of cancer from Zhongda Hospital, Nanjing, China (Table S1). This study was approved by the Ethics Committee of Zhongda Hospital, Southeast University.

### 4.2. Isolation of Plasma EVs 

Plasma was centrifuged at 300× *g* for 10 min, at 1200× *g* for 20 min, and at 10,000× *g* for 30 min at 4 °C to remove cells and debris. Then, EVs were purified by centrifugation at 200,000× *g* for 120 min and resuspended in PBS and purified by centrifugation at 100,000× *g* for 60 min. Immunoaffinity magnetic beads were used to isolate tumor-derived EVs as previously described [41,42]. After anti-EpCAM antibodies (Abcam, Cambridge, Mass, USA) were combined with magnetic beads (Invitrogen, Carlsbad, CA, USA), 3 μg of the anti-EpCAM beads were added to the extracted EVs followed by rotation overnight at 4 °C. A magnetic frame (Invitrogen, Carlsbad, CA, USA) was used to isolate the samples and obtain tumor cell-derived EVs.

### 4.3. Cell Culture

Human ESCC cell lines EC109, EC9706, and human monocytic leukemia cell line THP-1 obtained from the Key Laboratory of Environmental Medicine Engineering, Ministry of Education (Nanjing, China) were cultured in RPMI-1640 medium (Gibco, Carlsbad, CA, USA) supplemented with 10% EVs-free fetal bovine serum (FBS, Gibco, Carlsbad, CA, USA), 100 U/mL penicillin, and 100 U/mL streptomycin. A Transwell device (Corning, New York, NY, USA) with a 0.4 μm porous membrane was used for coculture treatments. EC109 or EC9706 cells transfected with miR-21-5p mimic/NC were seeded onto the upper chamber of the Transwell apparatus. THP-1 cells were seeded at a density of 2 × 10^5^ per well of the six-well plate. After 24 h of coculture, the cells were collected for further analysis. 

### 4.4. Macrophage Differentiation and Polarization

According to previous dosage experiments [43], 100 ng/mL phorbol myristate acetate (PMA, Sigma, Carlsbad, CA, USA) was used to induce the differentiation of THP-1 cells into M0 macrophages. M1 macrophages were obtained by incubating 100 ng/mL lipopolysaccharide (LPS, Sigma, USA) with M0 macrophages for 18 h. M2 macrophages were obtained by treatment with 20 ng/mL IL-4 (PeproTech, Bubendorf, Basel, Switzerland ) for 24 h.

### 4.5. Cell EVs Isolation and Identification

The EVs isolated from the supernatants of EC109 and EC9706 cells underwent ultracentrifugation as described in Section 4.2. We further confirmed that these vesicles were EVs by Western blot analysis of the tetraspanin molecules CD63 and TSG101 and the non-EVs protein GM-130. We also utilized a transmission electron microscope, HT7800 (Hitachi, Tokyo, Japan) to observe EVs according to the manufacturer’s instructions [44].

### 4.6. RNA Extraction and Quantitative Reverse Transcription PCR

TRIzol Reagent (Invitrogen, USA) was used to extract total RNA from cultured cells. The isolated total RNA was reversed transcribed using ReverTra Ace^®^ qPCR RT Master Mix with gDNA Remover (Toyobo, Osaka, Japan), and qRT-PCR analysis was performed with SYBR^®^Green Realtime PCR Master Mix (Toyobo, Japan). The miRNAs from plasma, cell supernatants and EVs were isolated with the miRNeasy Mini Kit (Qiagen, Shanghai, China) and reversed transcribed using MicroRNA Reverse Transcription Kit (RiboBio, Guangzhou, Guangdong, China). miRNA presence was quantified with Bulge-Loop^TM^ miRNA qRT-PCR (RiboBio, Guangzhou, Guangzhou, China). A NanoDrop 1000 spectrophotometer (Thermo Scientific, Carlsbad, CA, USA) was used to detect the total RNA concentration. Denaturing gel electrophoresis was used to explore RNA integrity. The mRNA level was normalized to that of β-actin, while the relative miRNA level was normalized to U6. All gene expression was analyzed by the StepOne System (Applied Biosystems, Carlsbad, CA, USA). The PCR primer sequences are shown in Table S2.

### 4.7. Enzyme-Linked Immunosorbent Assay (ELISA)

IL-6, TNF-α, IL-10, and TGF-β1 concentrations in the coculture system supernatant were detected with an ELISA kit (Yifeixue Biotech, Nanjing, Jiangsu, China) according to the manufacturer’s protocol [45].

### 4.8. Cy3-Labeled EVs-miR-21-5p Transfer Assay

The EVs were extracted from the supernatant of the EC109 cells transfected with a Cy3-miR-21-5p mimic and added to macrophages for 4 h. After blocking with an antifluorescence quenching sealer, we used a confocal microscope FV3000 (Olympus, Tokyo, Japan) to observe cell morphologies.

### 4.9. Flow Cytometry

Cells from samples of LPS- or IL-4- treated macrophages were suspended in PBS and incubated with anti-human CD86 APC and anti-human CD206 PE (Biolegend, San Diego, CA, USA) for 30 min at 4 °C in darkness. After incubation, cells were washed and analyzed using an FACSCalibur flow cytometer (BD Bioscience, Franklin Lake, NJ, USA).

### 4.10. Cell Transfection

To transfect the miRNA mimics and inhibitors, EC109 and EC9706 were seeded in six-well plates (3 × 10^5^/well) for 24 h. After the cells adhered to the well, 1.5 mL of RPMI-1640 medium without antibiotics were added to each well. Subsequently, these cells were transfected for 48 h with 20 nmol/L miR-21-5p mimic/inhibitor according to the Lipofectamine RNAiMAX (Invitrogen, Carlsbad, CA, USA) instructions.

### 4.11. Luciferase Activity Assay

THP-1 cells were cotransfected with wild-type (WT) or mutant (Mut) PTEN 3′UTR and miR-21-5p mimic or negative control using Lipofectamine 3000 (Invitrogen, USA). Cell lysates were harvested 48 h after transfection. We then measured firefly and *Renilla* luciferase activities by a dual-luciferase reporter assay kit (Promega, Madison, WI, USA) according to the manufacturer’s protocol. *Renilla* luciferase activity was used for normalization.

### 4.12. Western Blotting

EVs and cells were lysed with RIPA lysis buffer including protease and phosphatase inhibitors (Boyotime, Beijing, China). Protein concentrations were determined using the Pierce™ BCA Protein Assay kit (Thermo Scientific, USA). Western blots were performed as previously described [46]. The primary antibodies used in the experiments included anti-CD63 (1:500, Santa Cruz, CA, USA), anti-TSG101 (1:500, Santa Cruz, CA, USA), anti-GM-130 (1:500, Santa Cruz, CA, USA), anti-E-cadherin (1:1000, ABclonal, Wuhan, Hubei, China), anti-N-cadherin (1:1000, Affinity Biosciences, Cincinnati, OH, USA), anti-Snail (1:1000, ABclonal, Wuhan, Hubei, China), anti-α-SMA (1:1000, Cell Signaling Technology, Danvers, MA, USA), anti-PTEN (1:1000, Cell Signaling Technology, USA); anti-PI3K (1:1000, Abcam, USA), anti-Akt, anti-p-Akt (Ser473, 1:1000, Abcam, USA), anti-p-STAT6 (Y641, 1:1000, Abcam, USA), anti-p-Smad2 (1:1000, Abcam, USA), anti-GAPDH (1:1000, ABclonal, Wuhan, Hubei, China), and anti-β-actin (1:1000, Abcam, USA). ImageJ version 1.48 was used to analyze Western blot results [47]. 

### 4.13. Cell Migration and Invasion Assays

Cell migration and invasion assays were performed using 24-well plates and an 8 μm pore-size chamber (Corning, New York, NY, USA). For the migration assays, 2 × 10^5^ EC109 or EC9706 cells were seeded in the upper chamber with serum-free RPMI-1640 medium, and 600 μL of RPMI-1640 medium containing 10% FBS was added to the lower chamber. For the invasion assay, a Matrigel matrix (50 μL/well, BD Biosciences, USA) was added to the upper chamber. Approximately 5 × 10^5^ tumor cells were seeded on the upper chamber with serum-free RPMI-1640 medium, and 600 μL of RPMI-1640 medium containing 50% FBS was added to the lower chamber. After 24 h of coculture, EC109 or EC9706 cells were fixed with 95% ethanol for 15 min and stained with 0.1% crystal violet for 20 min. After removing nonmigrating or noninvasive cells, six visual fields were randomly chosen for calculations.

### 4.14. Statistical Analysis

All of the biological experiments were performed in triplicate. Statistical analyses were performed with SPSS software version 21.0. All data were presented as mean ± SD. Student’s *t*-test was used for comparisons of statistical significance. We compared more than two groups with one-way ANOVA. The significance level α was set at 0.05.

## 5. Conclusions

In conclusion, miR-21-5p was increased in patients with ESCC, and this miRNA contributed to the polarization of M2 macrophages in TAM. We found that EVs-miR-21-5p from EC109 or EC9706 cells were taken up by PMA-treated M0 macrophages which were then transformed to M2 macrophages. This contributed to the EMT process of esophageal cancer cells. The mechanism of these changes may be related to the PTEN/AKT/STAT6 pathway. This signaling causes high levels of TGF-β1 to be secreted by M2 macrophages. Our results indicate a positive feedback mechanism between M2 macrophage polarization and EMT of esophageal cancer cells in the TME via shuttling of tumor-derived EVs miR-21-5p.

## Figures and Tables

**Table 1:** Aberrant expression of EVs-miR-21-5p was associated with a high risk of esophageal carcinoma as determined by logistic regression analysis.

miRNA	Number of Samples	β	SE	Wald	*p*-Value	OR	95% CI
miR-21-5p	36	0.293	0.083	12.365	<0.001	1.340	1.138–1.578

**Table 2:** Diagnostic value of tumor-derived EVs-miR-21-5p in esophageal cancer.

miRNA	AUC	95% CI	Sensitivity	Specificity	Cut-Off
miR-21-5p	0.966	0.881–0.996	0.966	0.897	10.5

## Data Availability

Data is contained within the article or Supplementary Material.

## Supplementary Materials

The following are available online at https://www.mdpi.com/article/10.3390/cancers13164122/s1. Table S1 Basic characteristics of patients, Table S2. The sequences of primer pairs. The uncropped Western Blot images can be found in Supplementary Materials.

## Abbreviations

miRNAs	MicroRNAs
ESCC	Esophageal squamous cell carcinoma (ESCC)
EpCAM	Cell adhesion molecule
EVs	Extracellular vesicles
EMT	Epithelial-mesenchymal transition
TAMs	Tumor-associated macrophages
TME	Tumor microenvironment
OR	Odds ratio
