# Supplementary material for: Esophageal Cancer-Derived Extracellular Vesicle miR-21-5p Contributes to EMT of ESCC Cells by Disorganizing Macrophage Polarization
Source: Cancers (Basel). 2021 Aug 16;13(16):4122. doi: 10.3390/cancers13164122 (PMC8392810; doi:10.3390/cancers13164122)
Supplement: Supplementary file 1 [file cancers-13-04122-s001.zip › cancers-1240805-supp.pdf]

cancers

MDPI

*Article*

# Esophageal Cancer-Derived Extracellular Vesicle miR-21-5p Contributes to EMT of ESCC Cells by Disorganizing Macrophage Polarization

**Jing Song, Peiyan Yang, Xiuwen Li, Xinyi Zhu, Mengxin Liu, Xuexin Duan and Ran Liu**

**Table S1.** Basic characteristics of patients.

| Characteristics | ESCC, N (%) | Healthy Control, N (%) | *P*-Value |
|---|---|---|---|
| Total | 36 (100) | 36 (100) | |
| Age, Year | 60.14 ± 7.19 | 59.72 ± 5.36 | 0.781 |
| Gender (male/female) | 15/21 | 12/24 | 0.465 |
| TNM staging | | | |
| T1 | 9 (25.0) | | |
| T2 | 13 (36.1) | | |
| T3 | 14 (38.9) | | |
| T4 | 0 (0.0) | | |
| Lymph node metastasis | | | |
| Positive | 7 (19.4) | | |
| Negative | 29 (80.6) | | |

**Table S2.** The sequences of primer pairs.

| Gene | Forward Primer (5′–3′) | Reverse Primer (5′–3′) |
|---|---|---|
| CD68 | CTTCTCTCATTCCCCTATGGACA | GAAGGACACATTGTACTCCACC |
| IL-6 | CACTGGTCTTTTGGAGTTTGAG | GGACTTTTGTACTCATCTGCAC |
| TNF-α | CGTGGAGCTGGCCGAGGAG | AGGAAGGAGAAGAGGCTGAGGAAC |
| IL-1β | ATGATGGCTTATTACAGTGGCAA | GTCGGAGATTCGTAGCTGGA |
| IL-12 | TGCCCATTGAGGTCATGGTG | CTTGGGTGGGTCAGGTTTGA |
| SOCS3 | GAGGCTGGAGGTCATTGGAGAGG | AGGTAATTCCATCGCTGCTACATTCC |
| NF-kB p65 | CCCACGAGCTTGTAGGAAAGG | CCTGGTCCTGTGTAGCCATT |
| CCR7 | TGAGGTCACGGACGATTACAT | GTAGGCCCACGAAACAAATGAT |
| IDO1 | GCCAGCTTCGAGAAAGAGTTG | ATCCCAGAACTAGACGTGCAA |
| IL-10 | CCTGGAGGAGGTGATGCCCCA | CCTGCTCCACGGCCTTGCTC |
| TGF-β1 | CTCTGCCTCCTCCTGCCTGTC | AGAGTGTTGCTATGGTGACTGAATGAG |
| CD206 | CTACAAGGGATCGGGTTTATGGA | TTGGCATTGCCTAGTAGCGTA |
| CD209 | AATGGCTGGAACGACGACAAA | CAGGAGGCTGCGGACTTTTT |
| CD163 | TGTGGCCTGCATAGAGAGTG | TTCCCCAAAATGAGCAGAAC |
| CCL13 | CTCAACGTCCCATCTACTTGC | TCTTCAGGGTGTGAGCTTTCC |
| β-actin | CCACTGGCATCGTGATGGA | CGCTCGGTGAGGATCTTCAT |
| U6 | CTCGCTTCGGCAGCACA | AAACGCTTCACGAATTTGCGT |
| miR-21-5p | GCGCGTAGCTTATCAGACTGA | AGTGCAGGGTCCGAGGTATT |
| miR-193b | GCGAACTGGCCCTCAAAGT | AGTGCAGGGTCCGAGGTATT |
| let-7c-5p | CGCGCGTGAGGTAGTAGTTTGT | AGTGCAGGGTCCGAGGTATT |
| miR-181a-5p | CGAACATTCAACGCTGTCG | AGTGCAGGGTCCGAGGTATT |
| let-7i-5p | CGCGCGTGAGGTAGTAGTTTGT | AGTGCAGGGTCCGAGGTATT |
| miR-223-3p | GCGCGTGTCAGTTTGTCAAAT | AGTGCAGGGTCCGAGGTATT |
| miR-146a | CGCGTGAGAACTGAATTCCA | AGTGCAGGGTCCGAGGTATT |
| miR-124 | CGTAAGGCACGCGGTGAA | AGTGCAGGGTCCGAGGTATT |
